# Megakaryocyte–Platelet Immunometabolism in Leukemic Niche Remodeling

**DOI:** 10.3390/cancers18142321

**Published:** 2026-07-18

**Authors:** Hoyeop Baek, Kiwon Lee

**Affiliations:** Department of Bioscience and Biotechnology, Hankuk University of Foreign Studies, Yongin 17035, Republic of Korea; qorghduq7@hufs.ac.kr

**Keywords:** megakaryocyte heterogeneity, platelet extracellular vesicles, leukemic niche, inflammatory MK states, mitochondrial stress, preleukemic inflammation, bone marrow remodeling

## Abstract

Megakaryocytes (MKs) and platelets are increasingly recognized as active regulators of the bone marrow (BM) microenvironment rather than passive effectors of thrombopoiesis and hemostasis. Recent single-cell and lineage-tracing studies have established that megakaryopoiesis generates functionally heterogeneous populations, including immune-biased and niche-supporting subsets that shape hematopoietic stem cell (HSC) behavior, inflammatory tone, and vascular homeostasis. In leukemia, these regulatory circuits are systematically rewired to establish a marrow niche that suppresses normal hematopoiesis while sustaining leukemic stem cell (LSC) fitness through cytokine gradients, stromal remodeling, and direct cell-to-cell communication. In this focused review, we propose that the immune MK (iMK)–platelet axis is a central driver of leukemic niche remodeling. We discuss how iMK states arise under leukemic pressure, how MK heterogeneity encodes distinct niche instructions, and how platelet-derived extracellular vesicles (EVs) distribute inflammatory signals across the marrow and systemic circulation. Within this framework, we position mitochondrial stress outputs—such as reactive oxygen species (mtROS), mitochondrial DNA (mtDNA) release, metabolic rewiring, and mitochondria-containing EV secretion—not as isolated phenomena, but as mechanistic amplifiers embedded within the broader inflammatory and niche-regulatory programs of MKs and platelets. We further highlight preleukemic inflammatory states as an underappreciated entry point for therapeutic intervention, and propose three clinically actionable axes: inflammatory niche interruption, mitochondrial stress modulation, and platelet–leukemia communication blockade. This framework aligns with emerging concepts in MK heterogeneity, innate immune sensing, endothelial remodeling, and preleukemic signaling, and positions the MK–platelet axis as a promising therapeutic framework in leukemia-associated niche remodeling.

## Simple Summary

Megakaryocytes are increasingly recognized as active regulators of bone marrow remodeling in hematopoietic malignancies, extending their role far beyond platelet production. Recent studies show that abnormal megakaryocytes and platelets release inflammatory molecules and small extracellular particles that alter immune responses, blood vessel function, and communication between cells within the bone marrow. These changes create a microenvironment that protects leukemia cells while suppressing normal blood formation. In this review, we summarize recent advances in understanding how megakaryocytes and platelets contribute to leukemia progression and discuss emerging therapeutic strategies aimed at disrupting these interactions. Targeting this previously underappreciated cellular network may provide new opportunities to improve leukemia treatment.

## 1. Introduction

The concept that leukemia is simply a disease of uncontrolled blast proliferation has been replaced by a more nuanced view. Leukemia is increasingly recognized not only as a disease of cell-intrinsic transformation, but also as a disorder of bone marrow ecosystem remodeling [1,2]. In leukemic settings, structural, cellular, and molecular changes within the bone marrow (BM) niche suppress normal hematopoiesis, promote leukemic stem cell (LSC) survival, and sustain chronic inflammatory signaling that supports disease persistence and therapy resistance [1,2,3]. Among leukemic entities, acute myeloid leukemia (AML) has emerged as a prototypical model in which these niche-remodeling processes have been studied in greatest detail, and will therefore serve as the primary focus of this review.

Within the BM, megakaryocytes (MKs) and platelets occupy a uniquely strategic position. MKs sit at the vascular–stromal interface, where they integrate signals from the circulation, the endosteal zone, and resident immune cells [4,5]. They communicate with hematopoietic stem cells (HSCs) through direct contact and through secretion of cytokines and chemokines, while the massive production of platelets continuously seeds the local and systemic microenvironment with bioactive cargo capable of influencing vascular integrity, immune responses, and stromal behavior [4,5,6,7]. Platelets, in turn, are not simply hemostatic particles. They express a broad repertoire of pattern-recognition receptors, respond to tissue damage and microbial signals within seconds, and release extracellular vesicles (EVs) that carry proteins, lipids, nucleic acids, and metabolically active mitochondria to recipient cells throughout the body [6,7,8].

Historically, the literature on MKs and platelets in leukemia has focused on thrombocytopenia as a consequence of BM failure or on platelet-mediated tumor immune evasion [9,10,11,12,13]. Earlier studies, constrained by the tools available at the time, appropriately emphasized these aspects; however, newer single-cell, imaging, and niche-mapping studies now reveal a far more active role for the MK–platelet compartment. MKs and platelets do not merely reflect leukemic BM injury, but actively participate in shaping the leukemic niche. Recent single-cell transcriptomic analyses have demonstrated that MKs in human acute myeloid leukemia (AML)-bearing marrows are reprogrammed toward inflammatory states that differ fundamentally from their homeostatic counterparts [1,3,14,15]. Platelet-derived EVs in leukemic settings carry altered mitochondrial and inflammatory cargo that modifies stromal cell metabolism, endothelial function, and LSC survival signaling [6,16,17,18].

A key conceptual advance underlying this emerging view is the recognition that MKs are functionally heterogeneous. Recent single-cell and lineage-tracing studies have redefined megakaryocyte biology by demonstrating that the MK lineage contains functionally distinct subsets, including “classical” thrombopoietic MKs (tMKs) dedicated to platelet production, immune-biased MKs (iMKs) enriched for innate immune programs, and niche-supporting MKs (nMKs) that maintain HSC quiescence through CXCL4-, TGF-β-, and thrombopoietin (TPO)-dependent signaling. In AML and related myeloid malignancies, these subsets are not affected uniformly: inflammatory iMK states expand, whereas nMK-like quiescence-supporting functions are reduced, creating a marrow environment that simultaneously fails to sustain normal hematopoiesis and increasingly favors LSC protection. This imbalance between inflammatory iMKs and niche-supportive nMKs provides a mechanistic link between MK heterogeneity, leukemic niche remodeling, and disease progression.

**Figure 1:**
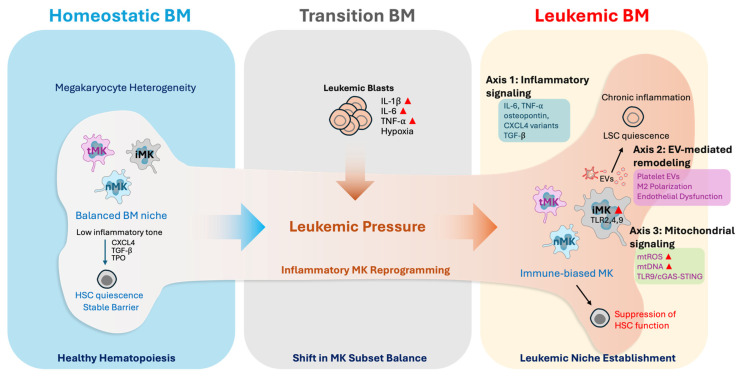
Integrated model of inflammatory megakaryocyte reprogramming and leukemic niche remodeling in AML. Under homeostatic conditions, functionally distinct MK subsets maintain balanced hematopoiesis and HSC quiescence through CXCL4-, TGF-β-, and TPO-dependent signaling. In this schematic, “transition BM” denotes an inflammatory, preleukemic marrow state (e.g., high-risk MDS, CHIP, RUNX1-familial platelet disorder) in which clonal HSCs and dysplastic/preleukemic blasts are present, but frank AML with a high blast burden has not yet emerged. Under leukemic stress, inflammatory cytokines and hypoxia promote expansion of iMK states and loss of niche-supportive functions. Reprogrammed MKs and platelets remodel the leukemic niche through inflammatory cytokine secretion, EV-mediated communication, and mitochondrial stress signaling, thereby suppressing normal hematopoiesis while supporting LSC survival, immune evasion, and therapy resistance. Abbreviations: MK, megakaryocyte; iMK, immune MK; HSC, hematopoietic stem cell; LSC, leukemic stem cell; BM, bone marrow; TPO, thrombopoietin; AML, acute myeloid leukemia.

We propose that this iMK–platelet axis deserves to be recognized as a central and currently underappreciated driver of leukemic niche remodeling. Although leukemic niche remodeling can occur across multiple myeloid neoplasms, the mechanistic framework developed in this review is grounded predominantly in AML, with selected reference to chronic myeloid leukemia (CML) and preleukemic myeloid disorders when they illuminate mechanisms relevant to AML biology. To provide a coherent structure, we organize the discussion into three interlocking axes (Figure 1), focusing on inflammatory MK and platelet programs, mitochondrial stress outputs, and platelet–leukemia communication. We further highlight preleukemic inflammatory states as a key entry point for intervention, particularly in conditions such as CHIP, MDS, MPNs, and RUNX1-familial platelet disorder. These three axes are not confined to a single section: Section 2 establishes the MK heterogeneity concept on which all three axes depend; Section 3 and Section 5 develop Axis 1 (inflammatory MK–platelet programs) in established AML and in preleukemic states, respectively; Section 4 develops Axis 2 (mitochondrial stress); and Section 6 translates Axes 1–3 into therapeutic strategies. This mapping is summarized in Figure 1.

## 2. Megakaryocyte Heterogeneity: A New Conceptual Foundation

### 2.1. From Homogeneous Thrombopoiesis to Functionally Distinct Subsets

Megakaryocytes arise from hematopoietic stem cells through a stepwise progenitor hierarchy, classically via megakaryocyte–erythroid progenitors, and, as recently shown, also through an HSC-proximal, megakaryocyte-biased progenitor route that bypasses several canonical intermediate stages [19,20]. Commitment to the MK lineage is marked by acquisition of the surface markers CD41 (ITGA2B) and CD42b (GP1BA), progressive loss of CD34, and endomitotic polyploidization as cells mature. These developmental and surface-marker features, together with the tMK/iMK/nMK-defining characteristics summarized in Table 1, provide the basis for identifying and distinguishing the MK subsets discussed throughout this review.

For decades, megakaryopoiesis was modeled as a unidirectional differentiation cascade culminating in polyploid MKs that shed platelets into BM sinusoids [4,19]. This model, while accurate for bulk thrombopoiesis, failed to account for the remarkable functional diversity now revealed by single-cell RNA sequencing and high-dimensional flow cytometry [20,21]. Recent single-cell and lineage-tracing studies have redefined megakaryocyte biology by demonstrating that the MK lineage contains functionally distinct subsets: (1) thrombopoietic tMKs dedicated to platelet production; (2) immune-biased iMKs that express myeloid innate immune programs and produce pro-inflammatory cytokines; and (3) niche-supporting nMKs that maintain HSC quiescence through TGF-β, CXCL4, and direct contact (Figure 1 and Table 1) [20,22,23,24].

This heterogeneity is not stochastic but shaped by microenvironmental cues, developmental origin, and transcriptional programs, giving rise to functionally distinct megakaryocyte subsets, including iMKs. Single-cell studies have identified iMK populations enriched in innate immune and myeloid-like transcriptional signatures, characterized by high expression of pattern-recognition receptors, including Toll-like receptors (TLRs) such as TLR2, TLR4, and TLR9, and the capacity to respond to inflammatory stimuli by producing IL-1β-rich secretomes [20,24]. nMKs are anatomically biased toward the endosteal zone and require von Willebrand factor (VWF), platelet factor 4 (CXCL4), and TPO signaling for maintenance [4,5]. Together, these data support a model in which MK differentiation yields parallel functional trajectories—thrombopoietic, immune-biased, and niche-supporting—that encode distinct instructions for the BM microenvironment (Table 1).

### 2.2. Leukemia Selectively Expands iMK States

In AML and chromic myeloid leukemia (CML), leukemic blasts and BCR-ABL expressing cells actively reshape the MK compartment. Blast-derived cytokines (IL-1β, TNF-α, GM-CSF) amplify iMK programs while suppressing the nMK state (Figure 1) [9,25]. The net result is a paradox: MKs become simultaneously more inflammatory and less capable of supporting normal hematopoiesis. iMKs in leukemia produce IL-6, TNF-α, osteopontin, and CXCL4 variants that sustain LSC quiescence, activate stromal fibroblasts, and create a chronic low-grade inflammatory state in the marrow (Figure 1) [1,3,15,26,27].

Single-cell AML datasets and high-dimensional flow cytometry analyses further indicate that transcriptional modules corresponding to iMK-like programs (enriched for TLRs, IL1B, TNF, and interferon-stimulated genes) are relatively expanded, whereas nMK-like signatures (enriched for CXCL4, TGFB1, and niche-regulatory factors) are reduced in AML compared with healthy BM. In integrated analyses, this skewed iMK/nMK balance correlates with inflammatory gene scores, cytopenias, and adverse clinical features, suggesting that MK state composition encodes marrow inflammatory tone and leukemic niche integrity rather than representing purely descriptive heterogeneity [1,3,9,14,15,26,27,28,29,30].

A particularly striking recent finding from murine CML models demonstrated that BCR-ABL^+^ CD41^+^CD150^+^ megakaryocyte-lineage cells develop a senescent phenotype characterized by excessive TGF-β secretion that directly increases LSC leukemogenic capacity [28]. These senescent MKs are resistant to imatinib and accumulate during disease progression, establishing a self-reinforcing loop between MK senescence and LSC protection [28]. Although these observations arise from CML rather than AML, they reinforce the broader concept that MK states can be durably reprogrammed toward inflammatory or senescent phenotypes that sustain leukemic niches across myeloid malignancies. The iMK subsets also produce EVs with a qualitatively distinct cargo from homeostatic MK-derived EVs, including higher levels of mitochondrial components, inflammatory proteins, and immunomodulatory RNA, a molecular profile tailored to sustain the leukemic niche (Figure 1) [6,16,18].

### 2.3. Megakaryocyte Heterogeneity as a Biomarker and Therapeutic Framework

Recognition of MK heterogeneity has important translational implications for both disease stratification and therapeutic intervention. In particular, the balance between inflammatory iMKs and nMKs may serve as a functional indicator of bone marrow inflammatory tone, niche integrity, and leukemic progression [20,22,23,24]. Across independent datasets, higher iMK/nMK ratios are associated with elevated inflammatory cytokine levels, reduced HSC quiescence signatures, and features of niche dysfunction in AML and preleukemic myeloid disease, supporting this balance as a biomarker of disease state rather than a purely static lineage descriptor [26,27,28]. Emerging evidence further suggests that iMK states are not merely biomarkers of disease evolution but active contributors to preleukemic niche remodeling.

In patients with RUNX1-familial platelet disorder, a recent study demonstrated that aberrant activation of the CD74 signaling axis promotes iMK programs and disrupts normal hematopoiesis in the preleukemic BM environment [29,30]. In that setting, CD74–MIF signaling sustains NF-κB-driven inflammatory loops in MKs, leading to an expansion of iMK-like states, altered platelet production, and progressive niche inflammation. Pharmacologic targeting of this pathway suppressed BM inflammation and partially restored hematopoietic function, providing direct evidence that iMK reprogramming is mechanistically linked to disease progression rather than representing a secondary bystander phenomenon [30]. The mechanistic basis of this pathway, including CD74-MIF signaling and its pharmacologic reversal, is detailed in Section 5.2. These findings establish a translational bridge connecting inherited MK dysfunction, inflammatory niche remodeling, and leukemic transformation, while also identifying the CD74 axis as a clinically actionable therapeutic target in preleukemic conditions [29,30].

Taken together, MK heterogeneity provides both a conceptual and practical framework for understanding leukemic niche remodeling. At a conceptual level, it explains how the same lineage can simultaneously generate thrombopoietic output, immune amplification, and stem cell guardianship under homeostatic conditions, yet be selectively skewed toward inflammatory and senescent states in AML. At a translational level, it suggests that monitoring iMK/nMK balance and MK inflammatory transcriptional programs could inform risk stratification, preleukemic surveillance, and selection of niche-targeted therapies that normalize MK states rather than solely targeting blasts.

## 3. The MK–Platelet Axis Actively Remodels the Leukemic Niche

### 3.1. iMK Secretome and HSC/LSC Regulation

Under homeostatic conditions, MK-derived CXCL4, TGF-β1, and TPO create a molecular milieu that promotes HSC quiescence within the bone marrow niche (Figure 1) [2,4,5]. MKs are frequently positioned adjacent to HSCs in both endosteal and perivascular regions, and emerging imaging and niche-mapping studies suggest that direct MK–HSC interactions cooperate with soluble niche factors to reinforce stem cell dormancy and retention [2,4,25]. Although the precise adhesion mechanisms remain incompletely defined, pathways involving VWF-, CXCL4-, and potentially DARC-associated interactions appear to contribute to this quiescence supportive niche architecture established in mouse models [4,5,25]. Consistent with these observations, experimental MK depletion in mice was achieved genetically via Pf4-Cre; iDTR-mediated diphtheria toxin ablation of MKs in vivo induced spontaneous HSC cycling and loss of quiescence, demonstrating that MK-derived signals function as essential nonredundant regulators of HSC homeostasis [5,31].

However, in leukemia, these regulatory mechanisms become selectively rewired. Inflammatory iMK populations produce elevated levels of IL-6, TNF-α, and other stress-associated cytokines that drive emergency myelopoiesis and impair normal HSC maintenance, while simultaneously secreting excessive TGF-β that preserves LSC quiescence and promotes therapy resistance (Figure 1) [1,3,9,15,25,26]. This differential niche regulation, characterized by inflammatory suppression of normal hematopoiesis coupled with protective signaling toward LSCs, provides a mechanistic explanation for the paradoxical coexistence of cytopenia and leukemic blast expansion in AML. In single-cell AML datasets, MK clusters enriched for iMK-like transcriptional programs display upregulation of IL6, TNF, TGFB1, and CXCL4 variants, whereas nMK-like modules are relatively diminished, further supporting a shift from “stem cell guardian” toward “immune amplifier” roles under leukemic pressure [26].

iMKs also alter the cytokine landscape in ways that favor myeloid-biased hematopoiesis and blast expansion. Elevated IL-6 levels promote myeloid progenitor expansion and sustain the pro-inflammatory marrow state [27]. High CXCL4 variant secretion displaces CXCL12/SDF-1 gradients, disrupting normal HSC retention signals while redirecting blast trafficking toward BM sinusoids. These secretome shifts are not simply bystander effects of global cytokine excess: single-cell analyses confirm that leukemic MKs display cell-autonomous transcriptomic reprogramming of cytokine production pathways that persists even when blast-derived signals are experimentally attenuated [1,3,15].

### 3.2. Platelet-Derived EVs as Niche-Remodeling Signals

Platelets release a diverse spectrum of EVs, ranging from small exosomes (50–150 nm) to larger ectosomes and microvesicles (100–1000 nm) [6,7]. Under leukemic conditions, platelet EV production is markedly increased as a consequence of chronic platelet activation induced by blast-derived inflammatory mediators such as ADP, IL-1β, thrombin, and oxidative stress signals [8,10]. Importantly, the molecular cargo of leukemia-associated platelet EVs differs substantially from that of homeostatic platelet EVs. Compared with homeostatic platelet EVs, leukemia-associated EVs contain enriched inflammatory and mitochondrial cargo, including oxidized proteins, mtDNA, and regulatory microRNAs. These altered EVs actively remodel stromal, endothelial, and immune compartments within the leukemic niche [8,10,16,17]. This composition likely reflects upstream inflammatory and mitochondrial reprogramming within leukemic MKs and activated platelets, positioning platelet EVs as downstream effectors of the iMK–platelet axis rather than isolated byproducts of platelet activation [6,16,17,18].

These EVs exert multilayered effects on the leukemic niche. They can increase endothelial permeability by delivering bioactive lipids and other factors that destabilize endothelial tight junctions, thereby creating a vascular barrier more permissive to leukemic cell egress; this is consistent with independent evidence that platelet EVs can directly modulate endothelial and lymphatic barrier integrity, albeit in a protective direction in non-leukemic contexts [32]. In parallel, leukemia- and niche-derived EVs promote polarization of bone-marrow macrophages toward immunosuppressive M2-like states, which are characterized by IL-4/IL-10-driven transcriptional programs and a metabolic shift toward mitochondrial oxidative phosphorylation and fatty-acid oxidation [33,34,35,36]. Such M2-polarized macrophages display reduced phagocytic clearance of leukemic blasts and enhance leukemic stem cell fitness, in part by providing prosurvival paracrine signals and by boosting blast mitochondrial metabolism.

A key recent finding demonstrates that M2-polarized marrow macrophages donate functional mitochondria to leukemic blasts, a process that simultaneously reduces macrophage phagocytic capacity and enhances blast metabolic fitness [37]. Platelet EVs contribute upstream to this cycle by driving M2 polarization, making platelet EV signaling an indirect enabler of macrophage-to-blast mitochondrial transfer. This mechanistic chain—platelet EVs → M2 macrophage polarization → mitochondrial donation to blasts—illustrates how Axis 1 inflammatory MK–platelet signaling connects to Axis 2 mitochondrial stress amplification and Axis 3 platelet–leukemia metabolic crosstalk within a single integrated niche-remodeling program.

### 3.3. Endothelial Remodeling and Blast Dissemination

Endothelial progenitor cells (EPCs) in AML-bearing marrows display profoundly impaired colony-forming capacity and reduced expression of hematopoietic supportive factors, accompanied by elevated ROS levels [38]. During remission, EPC function partially recovers alongside a decrease in ROS and restoration of niche-supportive capacity, suggesting that endothelial dysfunction is dynamically coupled with disease state rather than irreversible [38]. Platelet EVs containing mtROS and oxidized lipids directly drive this endothelial dysfunction by inducing endothelial NF-κB activation, inflammatory adhesion molecule expression (ICAM-1, VCAM-1), and vascular leakiness [39,40]. Paradoxically, inflammatory endothelial activation also contributes to blast dissemination. ICAM-1 and VCAM-1 upregulation facilitates blast–endothelial adhesion and transendothelial migration, increasing systemic blast trafficking [41]. Mitochondrially activated platelets further enhance blast extravasation by forming platelet–blast aggregates through P-selectin/PSGL-1 and GPIbα interactions [11,12,13]. These aggregates provide both physical immune shielding and metabolically supportive interactions for circulating leukemic blasts [11,12,13,16,17]. Within Axis 1, endothelial remodeling therefore operates as both a local niche-disruptive event that impairs normal hematopoiesis and a systemic gateway that facilitates blast dissemination and metastatic spread of AML cells.

### 3.4. Innate Immune Reprogramming and Immune Evasion

The leukemic BM innate immune compartment is systematically reprogrammed toward tumor tolerance [1,42,43]. Platelet- and MK-derived EVs are central to this reprogramming through two distinct molecular mechanisms. First, EV-delivered TGF-β and IL-10 suppress dendritic cell maturation, NK-cell cytotoxicity, and CD8^+^ T-cell effector function, directly weakening anti-leukemic adaptive immunity [6,18,42,43].

Beyond direct immune suppression, platelet–blast aggregates cloak leukemic cells from NK-cell recognition by downregulating activating ligands and providing a physical barrier to immune synapse formation [11,12,13]. S100A8/A9 proteins, prostaglandins, and metabolic cytokines released by platelet-activated macrophages further strengthen LSC fitness and reduce immunogenic cell death [34,38,44,45,46,47].

Collectively, these mechanisms establish a multilayered immune evasion architecture that is both platelet-dependent and platelet-amplified. Within the Axis 1 framework, innate immune reprogramming is therefore interpreted not as an isolated phenomenon, but as a downstream consequence of iMK secretome alterations, platelet EV cargo changes, and mitochondrial stress signaling that converge on macrophages, dendritic cells, NK cells, and T cells in the leukemic BM niche.

## 4. Mitochondrial Stress as an Embedded Amplifier of Niche Inflammation

### 4.1. Reframing Mitochondrial Rewiring Within the Inflammatory Axis

**Figure 2:**
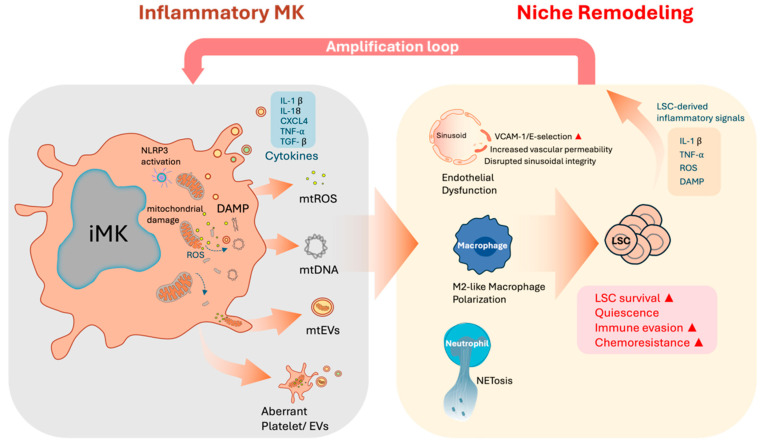
iMK reprogramming promotes mitochondrial amplification loops and leukemic niche remodeling. iMKs undergo profound mitochondrial and inflammatory reprogramming characterized by activation of the NLRP3 inflammasome and NF-κB signaling pathways, leading to enhanced secretion of pro-inflammatory cytokines, including IL-1β, IL-6, TNF-α, and IL-8. Dysfunctional mitochondria in iMKs generate elevated mtROS, release mtDNA, and produce mtEVs, establishing a self-amplifying inflammatory feedback loop. Aberrant platelet-derived extracellular vesicles further contribute to systemic inflammatory signaling. Within the leukemic bone marrow niche, these inflammatory mediators induce endothelial dysfunction, increase vascular permeability, and promote extracellular matrix remodeling. Mitochondrial danger-associated molecular patterns (DAMPs) and cytokine signaling drive M2-like macrophage polarization and NETosis, creating an immunosuppressive microenvironment that supports LSC survival. LSC-derived inflammatory signals further reinforce niche inflammation and chromatin instability, ultimately enhancing immune evasion, leukemic progression, and therapeutic resistance.

Rather than functioning as isolated metabolic abnormalities, mitochondrial stress pathways in MKs and platelets appear to reinforce and sustain inflammatory niche remodeling programs established during leukemic progression. Consistent with this view, a growing body of evidence has demonstrated widespread mitochondrial dysregulation across multiple leukemic cell populations and microenvironmental compartments [48,49,50]. LSCs exhibit high mitochondrial mass and dependence on oxidative phosphorylation (OXPHOS), while leukemic blasts display altered redox balance, defective mitophagy, and mitochondria-dependent metabolic adaptation associated with chemotherapy resistance [48,49,50]. Within the MK–platelet axis, mitochondrial stress acts less as a primary leukemogenic driver and more as a mechanistic amplification layer that magnifies inflammatory communication, prolongs niche remodeling, and deepens LSC protection, particularly when superimposed on Axis 1 inflammatory signaling (Figure 2) [48,49,50].

This reframing is supported by the logic of signal propagation in the leukemic niche. mtROS generated by iMKs and activated platelets does not merely damage cells: it activates NLRP3 inflammasomes, drives NF-κB signaling, and enhances cytokine production, thereby feeding back into the inflammatory programs already established by iMK subsets (Figure 2) [51,52,53]. Mitochondrial stress in MKs is transmitted to derived platelets through thrombopoiesis, seeding the circulating platelet pool with metabolically aberrant particles that have elevated basal ROS, exaggerated activation thresholds, and enhanced EV secretion. In this framework, mitochondrial outputs are best interpreted as integrated signaling modules embedded within inflammatory leukemic niche remodeling, rather than as stand-alone metabolic defects.

### 4.2. mtROS as an Inflammasome Trigger and Niche Remodeler

MKs under leukemic stress exhibit elevated mtROS arising from OXPHOS overactivation, impaired mitophagy, and accumulation of dysfunctional mitochondria (Figure 2) [48,49,50,51,52,53]. This mtROS elevation has three downstream consequences of niche relevance. First, it activates NLRP3 inflammasomes within MKs and platelets, driving IL-1β and IL-18 secretion that amplifies the local inflammatory tone in the BM (Figure 2) [51,52,53,54]. Second, elevated mtROS in MKs is transmitted to derived platelets through the thrombopoiesis process that mitochondria are directly incorporated into platelets during proplatelet formation, so MK mitochondrial dysfunction generates a population of metabolically aberrant platelets with constitutively elevated basal ROS, exaggerated activation thresholds, and enhanced EV secretion (Figure 2) [6,7,55,56]. Third, mtROS-driven oxidative modifications of platelet surface proteins and EVs alter their interaction with stromal cells, biasing EV uptake toward macrophages and endothelial cells that express scavenger receptors for oxidized lipids (Figure 2) [8,9,10,57].

These mtROS-dependent processes directly interface with Axis 1 by enhancing iMK secretome activation, promoting M2-like macrophage polarization, and aggravating endothelial dysfunction. They also set the stage for Axis 3 by generating platelet EV populations with increased propensity to transfer oxidatively stressed mitochondrial cargo to leukemic blasts.

### 4.3. The mtDNA–DAMP Axis: TLR9 and cGAS–STING

Platelets contain 2–10 copies of circular mitochondrial DNA per cell, releasable during strong activation, oxidative stress, gasdermin-D pore formation, or apoptosis-like mitochondrial permeability transition [58,59]. In leukemia, where platelets are chronically activated by blast-derived and stromal signals, basal mtDNA release is substantially elevated [60,61,62,63]. Extracellular platelet-derived mtDNA engages two major innate immune sensing pathways. Endosomal TLR9, expressed in pDCs, monocytes, and neutrophils, recognizes unmethylated CpG motifs in mitochondrial DNA and triggers pro-inflammatory type I interferon and IL-6/TNF-α production [60,61,62,63,64]. Cytosolic cGAS–STING, activated by mtDNA that escapes into the cytoplasm of stromal and immune cells, generates interferon-stimulated gene expression that enforces LSC quiescence and therapy resistance through STAT1 and NF-κB signaling [44,45,46,65,66,67,68]. Together, these pathways integrate platelet mitochondrial stress into BM innate immune circuits, producing an environment that is simultaneously cytokine-rich (elevated type I interferons, IL-6, TNF-α, neutrophil activation, and NETosis) and immunosuppressive (T-cell exhaustion, NK-cell dysfunction, and M2 macrophage polarization).

This paradoxical combination, which mirrors the “inflammatory but immunosuppressive” BM phenotype of AML, is mechanistically explained by the divergent downstream effects of TLR9 and STING activation in different BM cell types. Platelet-derived mtDNA may represent a convergent upstream inflammatory signal contributing to both immune activation and immunosuppressive remodeling within the leukemic marrow [60,63,67,69].

In the context of Axes 1 and 2, platelet-derived mtDNA can therefore be viewed as a convergent upstream DAMP that couples mitochondrial stress to innate immune reprogramming and LSC-protective signaling. Rather than treating TLR9 and cGAS–STING as separate stories, this review interprets them as coordinated sensors that translate platelet mitochondrial outputs into niche-level inflammatory and immunosuppressive remodeling (Figure 2 and Section 3.4).

### 4.4. Mitochondria-Containing EVs and Metabolic Cargo Transfer

A subpopulation of platelet-derived EVs retains functional mitochondria with intact electron transport chain complexes, membrane potential, and respiratory capacity [6,7,16,70,71,72,73,74]. These mitoEVs represent a distinct EV subclass capable of transferring metabolically active mitochondria to recipient cells, including neutrophils, tumor cells, and, by analogy, potentially leukemic blasts. Direct evidence for this transfer exists in platelet-to-neutrophil [70] and platelet-to-tumor-cell [71,72,74] systems; if a comparable mechanism operates in leukemic blasts, internalized platelet mitochondria would be expected to supplement endogenous respiratory capacity and increase OXPHOS output and ATP production, but direct demonstration of platelet-to-AML-blast mitochondrial transfer specifically is not yet available. This mitochondrial rescue is particularly relevant in the context of venetoclax-based therapy: venetoclax kills LSCs by disrupting OXPHOS through BCL-2 inhibition, and platelet-derived mitoEV transfer has been demonstrated in chronic lymphocytic leukemia (CLL), a related hematologic malignancy [75], and may, by extension, contribute to metabolic adaptation in AML, although this has not yet been directly tested in AML models.

The mechanistic chain linking platelet mitoEVs to venetoclax resistance has been partially validated in human CLL patient samples, where platelet microparticle internalization enhanced leukemic cell OXPHOS, reduced mitochondrial membrane potential consistent with mitochondrial uncoupling, and increased resistance to venetoclax and cytarabine [75,76]. In AML, the parallel biology of BMSC-to-blast mitochondrial transfer through tunneling nanotubes has been demonstrated to confer chemotherapy resistance [77]. Platelet mitoEV transfer likely operates as a second, rapidly renewable mitochondrial supply line given the massive platelet abundance in BM sinusoids, creating a metabolic redundancy that undermines OXPHOS-targeted therapies [6,7,16,70,71,72,73,74,75,76].

Within the Axis 2 framework, mitochondria-containing EVs thus function as mobile amplifiers that extend MK–platelet mitochondrial stress into leukemic blasts, macrophages, and stromal cells, reinforcing Axis 1 inflammatory remodeling and Axis 3 platelet–leukemia communication.

### 4.5. Mitophagy Impairment and the Dysfunctional Mitochondrial Pool

Under inflammatory and leukemic stress, MKs display impaired mitophagy resulting from dysregulation of the PINK1/Parkin axis and mTORC1 hyperactivation [48,49,50,51,52,53]. This impairment leads to the accumulation of damaged, depolarized mitochondria that produce excess ROS, release mtDNA, and generate abnormal metabolites (succinate, fumarate) with paracrine signaling capacity. The dysfunctional mitochondrial pool in iMKs is partially transferred to derived platelets, seeding the platelet population with mitochondria predisposed to excessive activation, EV shedding, and inflammatory cargo release. Restoring mitophagy flux in MKs through NAD^+^ boosting with NMN or NR, or through PINK1 activation, may normalize the downstream platelet mitochondrial stress program. Therefore, mitophagy restoration may serve as a proximal therapeutic intervention point with broad niche-normalizing effects [78,79,80,81,82]. In the Axis 2 context, such interventions are best conceptualized as upstream “reset” strategies that dampen mitochondrial stress amplification loops in MKs and platelets, thereby indirectly attenuating Axis 1 inflammatory signaling and Axis 3 platelet–leukemia metabolic support.

## 5. Preleukemic Inflammatory States: An Underappreciated Entry Point

### 5.1. MK Dysfunction in Preleukemic Conditions

The iMK–platelet axis is engaged early in disease evolution and appears to be an active driver, rather than a passive bystander, of preleukemic states [29,30]. Clonal hematopoiesis of indeterminate potential (CHIP), myelodysplastic syndromes (MDSs), and myeloproliferative neoplasms (MPNs) all involve inflammatory BM remodeling in which MKs and platelets play active roles [9,26,83,84,85,86]. In MPNs, JAK2V617F-driven MKs produce excess TGF-β and CXCL4 that drive BM fibrosis and suppress normal hematopoiesis long before overt leukemic transformation. In MDSs, dysplastic MKs generate platelet populations with impaired hemostatic function but enhanced inflammatory EV secretion, contributing to the chronic inflammatory marrow state that predisposes to AML transformation [1,6,16,18,83,84,85,86].

In CHIP, somatic mutations in TET2, DNMT3A, and ASXL1 promote inflammatory gene expression programs in myeloid cells, including MKs, through epigenetic derepression of NF-κB targets [84,85,86,87]. TET2-mutant MKs and platelets display elevated IL-6 and IL-1β output and abnormal EV cargo, establishing a pro-inflammatory BM environment that precedes and facilitates leukemic transformation. Across these entities, preleukemic MK and platelet dysfunction can be interpreted within Axis 1 as early skewing of the iMK/nMK balance, modest but persistent elevation of inflammatory cytokines, and qualitative changes in platelet EV cargo that progressively erode HSC-supportive niche functions while priming the marrow for leukemic colonization. These findings position iMK reprogramming as an early and potentially reversible driver of the leukemic trajectory, not merely a late consequence of established disease [30,87,88,89,90].

### 5.2. CD74 Signaling, RUNX1 Mutations, and Familial Platelet Disorder

A recent study demonstrated that targeting the CD74–MIF (macrophage migration inhibitory factor) axis in RUNX1-familial platelet disorder suppresses iMK programs, normalizes platelet production, and rescues defective hematopoiesis [30]. CD74 is a chaperone protein that moonlights as a receptor for MIF and D-DT, activating NF-κB and sustaining inflammatory cytokine loops in MKs. In the context of RUNX1 haploinsufficiency, which drives familial predisposition to AML, CD74 signaling is constitutively elevated in MKs, generating a chronic inflammatory niche that primes HSCs for transformation [30,91].

This study is particularly significant for our review because it provides proof-of-concept that the iMK state is not just a bystander but a functional driver of preleukemic hematopoietic failure, and that pharmacological reversal of MK inflammation can rescue the hematopoietic phenotype [30]. Within Axis 1, RUNX1-familial platelet disorder thus exemplifies how inherited MK dysfunction, CD74-dependent inflammatory signaling, and iMK expansion cooperate to create a preleukemic niche in which HSC quiescence is disrupted and leukemic risk is increased. The CD74 axis may therefore serve as both a biomarker of iMK activation and a therapeutic target in preleukemic conditions, offering an intervention window before overt AML emergence.

### 5.3. From Preleukemia to Established AML: A Continuous Inflammatory Trajectory

The preleukemic-to-leukemic transition can be understood as a progressive intensification of the same iMK–platelet circuit [29,30,83,84,85,86,87,88,89,90]. In early preleukemic stages, iMK subsets expand modestly, producing cytokine imbalances that create a mildly hostile environment for normal HSCs. nMK-like, quiescence-supporting MKs are still present but begin to lose dominance, and platelet EV cargo shows early signs of inflammatory and mitochondrial reprogramming. As clonal evolution proceeds and blast burden increases, iMK reprogramming deepens, platelet EV cargo becomes increasingly pathological, and the mtDNA–DAMP axis is more extensively activated (Figure 2) [6,16,18,60,61,62,63].

Once overt AML is established, the inflammatory bone marrow niche is fully entrenched: normal hematopoiesis is suppressed, the endothelium becomes dysfunctional, innate immunity is skewed toward tumor tolerance, and the mitochondrial stress amplification loop operates at maximal intensity (Figure 2) [1,33,34,35,36,37,38,39,40,41,42,43,60,64]. In this fully leukemic state, Axis 1 inflammatory MK–platelet signaling, Axis 2 mitochondrial stress outputs, and Axis 3 platelet–leukemia communication are tightly interwoven—iMK secretomes, platelet EVs, mtROS, mtDNA, mitoEVs, and platelet–blast aggregates cooperate to maintain LSC fitness, inhibit effective immune surveillance, and confer resistance to cytotoxic and targeted therapies.

This continuous trajectory has a critical therapeutic implication: targeting the MK–platelet inflammatory axis at preleukemic stages, before full niche consolidation, may be far more effective than attempting to dismantle an established leukemic niche [29,30,83,84,85,86,87,88,89,90,91,92,93,94,95,96,97]. Accordingly, Axis 1-directed interventions are particularly attractive in high-risk MDS, MPN, or CHIP contexts, where inflammatory MK and platelet programs are already abnormal but the leukemic niche is not yet “locked in”.

## 6. Therapeutic Opportunities: Three Actionable Axes

Instead of compiling an exhaustive list of potential targets, we outline three mechanistically grounded, clinically tractable therapeutic axes that emerge directly from the iMK–platelet axis framework. Each axis engages a distinct facet of leukemic niche remodeling, and they are inherently synergistic, such that simultaneous modulation of all three is anticipated to yield more durable niche normalization than any single-axis approach. The principal targets, representative agents, and mechanistic rationales across these therapeutic axes are summarized in Table 2.

### 6.1. Axis 1: Inflammatory Niche Interruption

Axis 1 targets the cytokine, chemokine, and innate immune circuits that sustain leukemic BM inflammation, suppress normal hematopoiesis, and maintain LSC-supportive signaling [1,3,9,26,63]. TGF-β pathway inhibition with galunisertib (LY2157299), an oral ALK5 inhibitor, counters MK- and platelet-derived TGF-β excess that suppresses normal HSCs while maintaining LSC quiescence; galunisertib has shown preclinical efficacy in AML and MDS models and hematologic improvement with acceptable safety in phase II lower-risk MDS studies, and its combination with hypomethylating agents could simultaneously reverse niche-mediated suppression and cell-intrinsic LSC protection [98,99,100,101]. Complementing this, blockade of the CD74–MIF axis suppresses iMK programs, normalizes platelet production, and rescues hematopoiesis, positioning ibudilast (MN-166) and anti-CD74 antibodies such as milatuzumab as attractive candidates in preleukemic and MDS settings [30,102]. Targeting IL-1β and IL-6 with agents such as canakinumab (anti–IL-1β) and tocilizumab (anti–IL-6R) directly suppresses key inflammatory cytokines amplified by NLRP3 inflammasome activity in iMKs. These agents have well-characterized safety profiles and are already under investigation in MDS and AML alongside standard therapies, with particularly strong rationale in preleukemic stages where cytokine-driven HSC suppression and iMK expansion are dominant features of niche pathology [103,104,105]. Finally, antagonism of TLR9 with agents like IRS954 blocks mtDNA-driven innate immune activation, thereby reducing pDC type I interferon production, NK-cell exhaustion, and M2 macrophage polarization, and is particularly suited for use alongside immune checkpoint blockade, where normalization of innate immune tone may potentiate T-cell-mediated anti-leukemia responses [106,107].

Within the Axis 1 framework, these interventions are best conceptualized as strategies that dampen iMK secretome activation, normalize MK/platelet-derived cytokine and chemokine gradients, and reset innate immune tone, thereby weakening the inflammatory scaffolding on which leukemic niche remodeling depends.

### 6.2. Axis 2: Mitochondrial Stress Modulation

Axis 2 aims to dampen mitochondrial stress outputs in MKs and platelets, thereby reducing mtROS-dependent inflammasome activation, mtDNA–DAMP signaling, and mitoEV-mediated metabolic support within the leukemic niche. Augmenting NAD^+^ levels with precursors such as NMN or NR can restore mitophagy flux in iMKs by activating PINK1/Parkin through NAD^+^-dependent SIRT1/SIRT3 signaling, thereby limiting dysfunctional mitochondria, reducing basal mtROS, and shifting the MK secretome toward a more homeostatic CXCL4/TGF-β ratio. The oral bioavailability and favorable toxicity profiles of NMN and NR provide a practical basis for testing these agents in early-phase AML and preleukemic clinical trials [108,109]. In parallel, mitochondria-targeted antioxidants such as MitoQ and SkQ1 selectively neutralize mtROS within MKs and platelets, attenuating NLRP3 inflammasome activation and reducing inflammatory EV cargo. MitoQ has reversed pro-thrombotic and pro-inflammatory platelet phenotypes in preclinical models, suggesting that similar approaches may normalize MK–platelet mitochondrial stress programming in leukemic context [110,111]. Pharmacological activation of PINK1/Parkin-mediated mitophagy represents an additional upstream lever: by enhancing mitochondrial quality control in MKs, such agents could reduce the generation of dysfunctional mitochondrial pools that feed Axis 2 amplification loops and downstream Axis 3 mitoEV transfer. Downstream of these upstream modulators, STING antagonists such as H-151 could in principle be repurposed to counter cGAS-STING-driven interferon-stimulated-gene immunosuppression in the leukemic niche [112,113,114], while OXPHOS-targeted agents such as IACS-010759 limit the mitochondrial metabolic rescue that sustains LSCs and may restore venetoclax sensitivity in combination regimens [115,116].

Taken together, Axis 2 strategies function as metabolic dampeners that attenuate mitochondrial stress amplification circuits in MKs and platelets. In doing so, they indirectly weaken Axis 1 inflammatory niche remodeling and Axis 3 platelet–leukemia metabolic support, providing a complementary layer of niche normalization that may enhance the durability of cytokine-directed therapies.

### 6.3. Axis 3: Platelet–Leukemia Communication Blockade

Axis 3 focuses on disrupting direct platelet–leukemia interactions, including EV-mediated cargo transfer, platelet–blast aggregate formation, and mitochondrial exchange, which collectively reinforce immune evasion and metabolic adaptation. EV biogenesis inhibitors such as neutral sphingomyelinase (nSMase2) inhibitors can reduce the production of platelet-derived EVs and mitoEVs, limiting the dissemination of inflammatory and mitochondrial cargo to leukemic blasts, macrophages, and stromal cells [117]. In parallel, blockade of adhesion pathways that underlie platelet–blast aggregate formation, such as P-selectin/PSGL-1 and GPIbα–von Willebrand factor interactions, can dismantle platelet cloaking of leukemic cells, thereby enhancing NK-cell recognition, reducing immune shielding, and impairing blast extravasation [11,12,13,118,119].

Targeting tunneling nanotube (TNT) formation and function, for example through modulation of connexin 43 or cytoskeletal regulators using agents such as carbenoxolone or the CX43-selective peptide Gap27, represents a complementary strategy to limit direct mitochondrial transfer from BM stromal cells or MK–platelet compartments to leukemic blasts [120,121,122]. By restricting these physical conduits, TNT-targeted interventions may reduce OXPHOS reinforcement and attenuate resistance to venetoclax and cytarabine observed in preclinical models. Given the emerging data linking platelet mitoEV internalization to venetoclax resistance in related hematologic malignancies, combining Axis 3 interventions with BCL-2 inhibition and hypomethylating agents may help neutralize metabolic escape routes and enhance LSC eradication [70,71,72,73,74,75,76].

Within the integrated framework of Axes 1–3, platelet–leukemia communication blockade is therefore positioned as a niche-directed adjunct that prevents MK–platelet signals from being converted into blast-level metabolic and immunologic advantages. Together with inflammatory niche interruption and mitochondrial stress modulation, Axis 3 interventions support a multi-layered strategy aimed at dismantling leukemic niche remodeling at both microenvironmental and leukemic-cell interfaces.

## 7. Conclusions

Myeloid leukemias are increasingly recognized as a disease in which cell-intrinsic oncogenic programs and BM microenvironment remodeling cooperate to sustain malignant hematopoiesis. Within this broader view, MKs and platelets have emerged as active regulators rather than passive bystanders of leukemic niche remodeling. The concept of MK heterogeneity with thrombopoietic tMKs, immune-biased iMKs, and niche-supporting nMKs provides a useful framework to understand how the same lineage can simultaneously support normal hematopoiesis under homeostatic conditions yet be selectively skewed toward inflammatory and senescent states in AML and preleukemic myeloid disorders.

In this review, the iMK–platelet axis is positioned along three interlocking therapeutic and mechanistic axes. Axis 1 describes inflammatory MK and platelet programs that remodel the leukemic niche by altering HSC and LSC regulation, stromal and endothelial behavior, and innate and adaptive immune tone. Axis 2 interprets mitochondrial stress, including mtROS, mtDNA release, mitophagy impairment, and mitochondria-containing EVs, as an embedded amplifier of these inflammatory circuits in MKs and platelets. Axis 3 focuses on direct platelet–leukemia communication, including EV-mediated cargo transfer, platelet–blast aggregates, and mitochondrial exchange, which collectively reinforce immune evasion and metabolic adaptation. Viewing leukemic niche remodeling through these three axes helps integrate diverse strands of MK, platelet, immune, and metabolic biology into a coherent, clinically actionable framework.

A central conclusion from this synthesis is that MK and platelet dysregulation begins early in preleukemic conditions such as CHIP, MDS, MPN, and RUNX1-familial platelet disorder, and progressively intensifies as clonal evolution proceeds toward established AML. Preleukemic MKs and platelets already exhibit skewed iMK/nMK balance, elevated inflammatory cytokine output, and altered EV cargo, generating a BM environment that suppresses normal HSC function and primes the niche for leukemic colonization. By the time that overt AML is established, these processes coalesce into a fully entrenched inflammatory and metabolically amplified leukemic niche characterized by cytopenias, endothelial dysfunction, innate immune tolerance, and therapy-resistant LSC pools. This continuous trajectory emphasizes that the iMK–platelet axis is not only a late disease feature, but an early and potentially reversible driver of leukemic evolution.

From a translational perspective, three major future directions emerge naturally from this schema. First, systematic single-cell and spatial profiling of MK and platelet states across preleukemic and AML cohorts will be essential to define clinically robust MK-based biomarkers, including iMK/nMK ratios, MK transcriptional modules, and platelet EV signatures, and to link these to inflammatory scores, niche integrity, and treatment outcomes. Second, mechanistic studies using primary AML samples and relevant models should further dissect how mitochondrial stress in MKs and platelets couples to mtROS-driven inflammasome activation, mtDNA–TLR9/cGAS–STING signaling, and mitoEV-mediated metabolic support, particularly in the context of venetoclax-based and OXPHOS-targeted therapies. Third, early-phase clinical trials are needed to test axis-directed combination strategies, including inflammatory niche interruption, mitochondrial stress modulation, and platelet–leukemia communication blockade in both preleukemic and AML settings.

Ultimately, the leukemic BM niche is shaped not only by blast-intrinsic genetic alterations, but also by dynamic, inflammatory, and mitochondrial reprogramming of MKs and platelets. Recognizing the iMK–platelet axis as a biologically relevant and therapeutically actionable component of AML pathophysiology opens an opportunity to complement traditional blast-centric approaches with niche-focused interventions. Such strategies may be particularly powerful when applied early, in high-risk preleukemic states, where the leukemic niche is not yet fully consolidated and MK/platelet programs remain partially reversible. Targeting the MK–platelet axis along the three proposed axes—before and after AML onset—offers a promising path toward deeper and more durable remissions by simultaneously dismantling leukemic stem cell support, normalizing BM inflammatory tone, and restricting platelet-mediated metabolic and immune advantages. Although the title refers to leukemic niche remodeling more broadly, the mechanistic evidence synthesized here is grounded predominantly in AML, and extrapolation of this framework to other myeloid or lymphoid leukemias should be made with appropriate caution until directly tested in those settings.

## Figures and Tables

**Table 1:** Functional heterogeneity of megakaryocytes and their roles in HSC regulation.

Feature	Thrombopoietic MK (tMK)	Immune MK (iMK)	Niche MK (nMK)	Key References
Primary function	Platelet production and release	Immune modulation and inflammatory signaling	Maintenance of HSC quiescence	[19,20,21]
Localization	Adjacent to bone marrow sinusoids	Distributed throughout BM; enriched during inflammation	In proximity to HSC niche (endosteal/perivascular regions)	[4,5]
Morphological features	Highly polyploid; extensive proplatelet formation	Smaller size; secretory phenotype	Sessile; closely associated with HSCs	[20,21]
Key markers/genes	VWF, ITGA2B(CD41), GP1BA, CXCL4	IL-1B, TNF, TLRs, interferon-stimulated genes	CXCL4, TGFB1, niche regulatory factors	[20,21,22,23,24]
Dominant signaling pathways	TPO–MPL–JAK2–STAT axis	TLR-NF-κB and interferon signaling	TGF-β-SMAD, CXCL4-mediated suppression	[2,4,5,19,20,25]
Functional output	Platelet biogenesis and release into circulation	Cytokine secretion, immune cell recruitment and activation	Suppression of HSC proliferation; maintenance of quiescence	[4,5,19]
Impact on HSCs	Indirect (via platelet-derived signals)	Promotes HSC activation and potential exhaustion under stress	Directly enforces HSC quiescence and retention	[2,4,5,25]
Response to inflammation	Reduced platelet output or functional impairment	Expanded population; dominant phenotype	Reduced HSC-supportive capacity	[9,24,26]
Alteration in leukemia/preleukemia	Decreased or dysfunctional platelet production	Skewed toward inflammatory programs; supports leukemic niche	Loss of quiescence-supporting function	[1,3,9,14,15,26,27,28]
Conceptual role	“Platelet factory”	“Immune amplifier”	“Stem cell guardian”	

Abbreviations: MK, megakaryocyte; tMK, thrombopoietic MK; iMK, immune MK; nMK, niche-supporting MK; HSC, hematopoietic stem cell; TPO, thrombopoietin; VWF, von Willebrand factor; CXCL4, platelet factor 4; TGFB1, transforming growth factor beta 1; TLR, Toll-like receptor. Subsets were defined primarily by single-cell RNA sequencing and high-dimensional flow/mass cytometry (CyTOF) of human and murine bone marrow, as detailed in the studies cited in the Key References column.

**Table 2:** Summary of therapeutic targets within the iMK–platelet axis.

Axis	Target	Agent/Strategy	Rationale
Axis 1	TGF- β pathway	Galunisertib (LY2157299)	Reverse MK-driven LSC quiescence normalize HSC suppression; Ph II ongoing in MDS/AML
Axis 1	CD74-MIF axis	Ibudilast; anti-CD74 (milatuzumab)	Suppress iMK program in preleukemic states; rescue hematopoiesis
Axis 1	IL-1β/IL-6	Canakinumab; tocilizumab	Block NLRP3 inflammasome cytokine output; reduce marrow inflammatory tone
Axis 1	TLR9/mtDNA	IRS954 (TLR9 antagonist)	Block pDC type I IFN; restore NK/T-cell anti-leukemic function
Axis 2	Mitophagy flux	NMN, NR (NAD+ precursors)	Restore PINK1/Parkin pathway; reduce dysfunctional MK mitochondrial pool
Axis 2	MtROS/NLRP3	MitoQ, SkQ1	Dampen inflammasome activation; normalize platelet EV inflammatory cargo
Axis 2	cGAS-STING	H-151 (STING antagonist)	Counter ISG immunosuppression; complement checkpoint inhibitor therapy
Axis 2	OXPHOS rescue	IACS-010759+ venetoclax	Limit mitochondrial-mediated metabolic rescue; restore venetoclax sensitivity
Axis 3	Platelet–blast adhesion	Crizanlizumab (anti-P-selectin); anti-GPIbα	Reduce platelet-mediated immune protection of blasts; restore NK cytotoxicity
Axis 3	EV biogenesis	GW4869 (nSMase2 inhibitor)	Reduce platelet mitoEV and inflammatory EV output to blasts and stroma
Axis 3	Tunneling nanotubes	Carbenoxolone; Gap27 (CX43 inhibitor)	Block TNT-mediated mitochondrial transfer; prevent LSC metabolic rescue

## Data Availability

No new data were created or analyzed in this study. Data sharing is not applicable to this article.
